# Investigating the Mutagenicity of a Cold Argon-Plasma Jet in an HET-MN Model

**DOI:** 10.1371/journal.pone.0160667

**Published:** 2016-09-01

**Authors:** Susanne Kluge, Sander Bekeschus, Claudia Bender, Hicham Benkhai, Axel Sckell, Harald Below, Matthias B. Stope, Axel Kramer

**Affiliations:** 1 Institute of Hygiene and Environmental Medicine, University Medicine Greifswald, Walther-Rathenau-Str. 49a, 17485 Greifswald, Germany; 2 Leibniz-Institute for Plasma Science and Technology, ZIK *plasmatis*, Felix-Hausdorff-Str. 2, 17489 Greifswald, Germany; 3 Department of Trauma and Reconstructive Surgery, University Medicine Greifswald, Sauerbruchstr., 17475 Greifswald, Germany; 4 Department of Urology, University Medicine Greifswald, Sauerbruchstr., 17475 Greifswald, Germany; Universite Toulouse III Paul Sabatier, FRANCE

## Abstract

**Objective:**

So-called cold physical plasmas for biomedical applications generate reactive oxygen and nitrogen species and the latter can trigger DNA damage at high concentrations. Therefore, the mutagenic risks of a certified atmospheric pressure argon plasma jet (kINPen MED) and its predecessor model (kINPen 09) were assessed.

**Methods:**

Inner egg membranes of fertilized chicken eggs received a single treatment with either the kINPen 09 (1.5, 2.0, or 2.5 min) or the kINPen MED (3, 4, 5, or 10 min). After three days of incubation, blood smears (panoptic May-Grünwald-Giemsa stain) were performed, and 1000 erythrocytes per egg were evaluated for the presence of polychromatic and normochromic nuclear staining as well as nuclear aberrations and binucleated cells (hen’s egg test for micronuclei induction, HET-MN). At the same time, the embryo mortality was documented. For each experiment, positive controls (cyclophosphamide and methotrexate) and negative controls (NaCl-solution, argon gas) were included. Additionally, the antioxidant potential of the blood plasma was assessed by ascorbic acid oxidation assay after treatment.

**Results:**

For both plasma sources, there was no evidence of genotoxicity, although at the longest plasma exposure time of 10 min the mortality of the embryos exceeded 40%. The antioxidant potential in the egg’s blood plasma was not significantly reduced immediately (*p* = 0.32) or 1 h (*p* = 0.19) post exposure to cold plasma.

**Conclusion:**

The longest plasma treatment time with the kINPen MED was 5–10 fold above the recommended limit for treatment of chronic wounds in clinics. We did not find mutagenic effects for any plasma treatment time using the either kINPen 09 or kINPen MED. The data provided with the current study seem to confirm the lack of a genotoxic potential suggesting that a veterinary or clinical application of these argon plasma jets does not pose mutagenic risks.

## Introduction

Transportable cold physical plasma sources, operating in the range of the body temperature (so-called cold plasma), pose new therapeutic options in medicine. For example, high potential is seen in the treatment of chronic wounds [1–3]. The kINPen cold plasma source used in this study has been shown to generate reactive oxygen and nitrogen species (ROS/RNS) in the gas phase [4–6] that diffuse into and react with liquids [7–9] and cells [10–12]. Thus, the oxidation of proteins and lipids is considered to be the plasma’s main route of action[13]. At low concentrations, these species contribute to cell signaling [14]. At high concentrations, they are cytotoxic, effectively inducing apoptosis [15–17]. Importantly, permanent and/or excessive oxidative stress, such as smoking or extensive exposure to UV-light, is known to be mutagenic [18]. It is therefore important to understand the genotoxic risks of plasma-derived ROS/RNS before plasma can be offered as standard therapy in clinics.

The high reactivity of oxygen radicals has two reasons. Thermodynamically, they are oxidizing agents. Kinetically, oxygen radicals and many other charged gas radicals undergo one-electron reactions which are much faster than more complex redox reactions [19]. As such, highly reactive species, for example hydroxyl radicals, even oxidize seemingly inert materials such as elemental gold [20]. However, vertebrate systems are equipped with an arsenal of options for ROS/RNS detoxification [21]. Yet, this defense system can be overcome by high concentrations of reactive molecules and some *in vitro* studies suggested DNA damage to be present following exposure to cold plasmas [22–29]. Yet, final conclusions of these studies using non-OECD tests are somewhat limited. First, they were carried out only in cell culture models. Second, they utilized read-out system such as cytochrome C release, Comet-assay, or the ATM/ATR system that are not exclusively linked only to DNA-damage but also to general oxidative stress and apoptosis [30–32]. By contrast, mutagenicity studies carried out and according to OECD guidelines concluded a lack of permanent DNA damage following exposure to cold plasma [33, 34], also for the plasma jet used in this study [35]. Nonetheless, all studies mentioned were *in vitro* work, and such test models do not allow for biologically relevant conclusions in vertebrate organisms.

We utilized fertilized chicken eggs and well-described mutagenic assays of blood cells to investigate the genotoxic hazard in these eggs following exposure to two atmospheric pressure argon plasma jets. The kINPen MED was approved as a class IIa medical device. Its use is indicated for the treatment of chronic or infected wounds as well as pathogen-related diseases of the skin. Its predecessor, the kINPen 09, has previously been used for veterinary purposes and differs in terms of energy output. Using the highly-sensitive chorioallantoic membrane of fertilized chicken eggs both jets have been shown to be tolerated well, suggesting the plasma’s compatibility with tissues [36]. Studies using porcine and human tissues underlined this notion [37, 38] which was a prerequisite for first observational studies in humans [2]. To complement the risks assessment made with the kINPen MED and kINPen 09, we here investigated their genotoxic potential.

## Materials and Methods

### The HET-MN model

**Fig 1:**
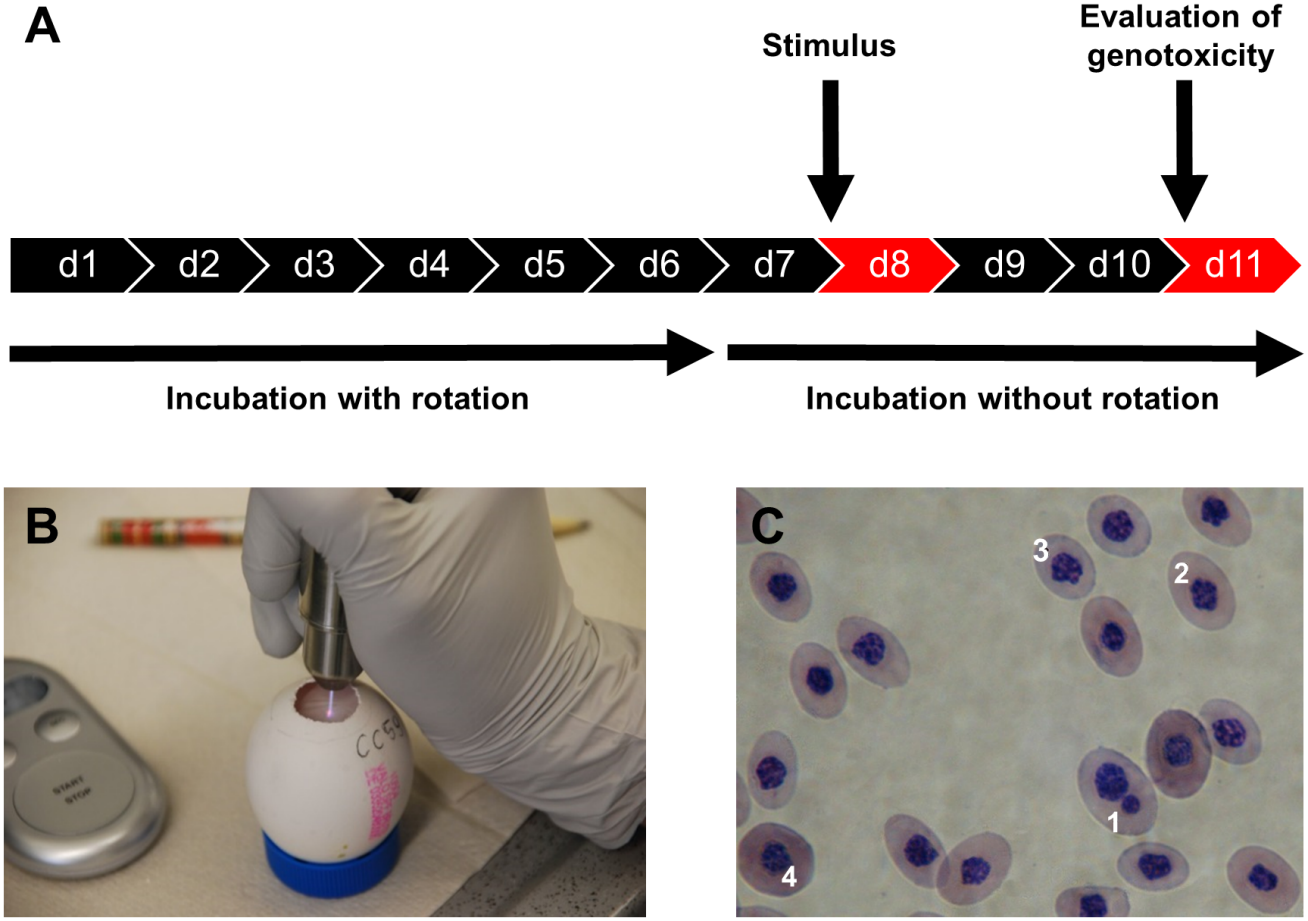
Cold plasma treatment of the HET-MN. (**A**) The experimental chronology is shown. (**B**) Treatment of the inner egg membrane with the kINPen MED. (**C**) A representative blood smear and Giemsa staining is shown. Labeling refers to micronucleated (1), normochromic (2), late polychromatic (3), and primitive (4) erythrocytes.

Due to lack of pain perception until day 11 of incubation [39], the hen’s egg test for micronucleus induction (HET-MN) is not classified as an animal experiment [40]. Fertilized, pathogen-free eggs were taken from Leghorn chickens (Lohmann Tierzucht, Germany). Eggs with intact shells were selected, weighted, and disinfected with 70% ethanol. Subsequently, eggs were incubated at 37.5 ± 1.0°C with a relative humidity of 62.0 ± 7.5% in a thermal incubator (J. Hemel Brutgeräte, Germany). From day one to day six, eggs were rotated automatically in a three-hour interval (Fig 1A). On day seven, they were placed in a vertical position with the blunt pole on top and further incubated without rotation. Following recent suggestions [41], the application of the test substances was carried out on day eight while blood was collected on day 11. On day eight, eggs were screened with a Powerlux-Lamp (Schier-lamp, J. Hemel Brutgeräte, Germany) for presence of blood vessels, and unfertilized eggs were sorted out. Immediately before opening the egg shell, the eggs were disinfected on the blunt pole to prevent penetration of microorganisms during preparation of the egg [42] because infection has been linked to increase embryo mortality [43]. Using the Schier-lamp, the boundaries of the air chamber were marked with a pen to prevent any violation of the inner shell membrane during the preparation process. The egg shell was carefully removed under aseptic conditions to uncover the inner egg membrane which was chosen for cold plasma application because it is considered to be very sensitive [32]. After exposure to the test agents, the egg opening was covered with a bacteria-impermeable, sterile, transparent *Tegaderm* dressing (3M, Germany), and incubated for three days before blood was collected to determine genotoxic effects. At the same time, embryo mortality was assessed.

### Cold plasma jets

Two atmospheric pressure argon plasma jets, the kINPen 09 and the kINPen MED (both neoplas, Germany), were tested. These sources were operated at a feed gas flow rate of 5 standard liters of argon per minute. In contrast to the pulsed (on:off cycle = 1:1) operation of the kINPen MED, the kINPen 09 generates its plasma in a continuous mode, resulting in higher energy densities. Operation details and parameters were previously described [44]. The gas temperatures at the typical working distance (8 mm effluent length) were 45°C for the kINPen 09 [45] and 37°C for the kINPen MED [46], respectively. Treatment was carried out manually. To ensure that during exposure the distance of either of the jets was not lower than 8 mm, an autoclavable spacer was used.

### Exposure of the inner shell membrane to cold plasma or test agents

The eggs were exposed on day eight. A 72 h contact time was allowed for all substances because it yields an high MNE II-rate, a low toxicity, and a high xenobiotic metabolisms [41, 47, 48]. Moreover, we wanted to investigate the long-term effects of plasma by identifying possible mutagenic effects of a single plasma exposure regimen which exceeded the recommended application time by far [1, 46, 49]. Prior to plasma exposure (Fig 1B) and to reduce dehydration, 100 μl of 0.9% NaCl solution was applied. To account for any effect of the NaCl application alone, the solution was added to the inner membrane also immediately after (instead of before) exposure to plasma for one treatment condition (5 min, kINPen MED). Exposure times for the kINPen 09 (30 s per spot) were 1.5 min (3 spots), 2.0 min (4 spots), or 2.5 min (5 spots), and for the kINPen MED (60 s per spot) 3 min (3 spots), 4 min (4 spots), 5 min (5 spots) or 10 min (10 spots). Spots were treated manually by holding the jet steady above each spot for 30 s (kINPen 09) or 60 s (kINPen MED) without any movement and with the visible tip of the effluent touching the egg’s surface (about 10 mm for the kINPen MED and 11 mm for the kINPen 09 but not less than 8 mm which was achieved by using an autoclavable spacer). Spots were treated one after the other with the spots being 5-10 mm apart from each other with the total treatment area being about 2 cm^2^. Several spots were treated to prevent perforation of the sensitive egg membrane, and different treatment times per spots (30 s for the kINPen 09 and 60 s for the kINPen MED) were chosen according to the plasma’s intensity (continuous mode in the kINPen 09 vs. pulsed operation in the kINPen MED). Only the visible plasma effluent was in contact with the inner egg’s membrane (Fig 1B). Positive controls were exposed to 100 μl PBS containing either 50 μg/egg of CYP450-activated, pro-mutagenic cyclophosphamide (CAS 50-18-0; Baxter Oncology, Germany) [50], or 5μg/egg of CYP450-independent, pro-mutagenic methotrexate (CAS 59-05-2; TEVA, Germany) [51]. Negative controls were exposed to either 100 μl 0.9% NaCl alone or non-ionized argon gas (flow rate of 5 standard liters per minute). After exposure, eggs were continued to be incubated for 3 days without rotation. Eleven independent experiments were conducted with three eggs per treatment group and experiment (1.5, 2.0, 2.5 min with the kINPen 09; 3, 4, and 5 min with the kINPen MED; NaCl negative control; 5 min of argon gas alone; and cyclophosphamide positive control). Within these eleven experiments, eight experiments also included 10 min (with NaCl addition before treatment) and 5 min (with NaCl addition after treatment) of kINPen MED plasma exposure with three eggs per group and experiment. Three experiments included 10 min treatment of argon gas, and seven experiments included treatment with methotrexate, both with three eggs per group and experiment. The total number of eggs among all experimental groups is given in Table 1 with differences to the planed number of eggs being attributed to sorting out eggs that were either unfertilized or damaged during the preparation procedure.

**Table 1:** Frequencies of nuclear aberrations, binucleated cells, and micronucleated definite erythrocytes.

Test Agent	kINPen Plasma (min)	Eggs (n)	Nuclear aberrations (‰)	Binucleated cells (‰)	micronucleated erythrocytes (‰):		
					polychromatic	normochromic	sum
kINPen 09	1.5	30	0.00 ± 0.00	0.30 + 0.79	0.03 + 0.18	0.13 + 0.35	0.17 + 0.38
	2.0	30	0.00 ± 0.00	0.29 ± 0.66	0.11 ± 0.31	0.18 ± 0.39	0.29 ± 0.46
	2.5	30	0.11 ± 0.31	0.29 ± 0.53	0.04 ± 0.19	0.21 ± 0.50	0.25 ± 0.52
kINPen MED (NaCl before treatment)	3	29	0.00 ± 0.00	0.61 ± 1.77	0.11 ± 0.31	0.29 ± 0.53	0.39 ± 0.63
	4	29	0.07 ± 0.26	0.46 ± 1.04	0.00 ± 0.00	0.14 ± 0.36	0.14 ± 0.36
	5	29	0.00 ± 0.00	0.17 ± 0.38	0.08 ± 0.28	0.17 ± 0.38	0.25 ± 0.44
	10	19	0.00 ± 0.00	0.36 ± 1.21	0.09 ± 0.30	0.18 ± 0.40	0.27 ± 0.47
kINPen MED (NaCl after treatment)	5	19	0.00 ± 0.00	0.24 ± 0.56	0.12 ± 0.33	0.24 ± 0.44	0.35 ± 0.49
NaCl	-	30	0.00 ± 0.00	0.17 ± 0.38	0.00 ± 0.00	0.30 ± 0.70	0.30 ± 0.70
Argon gas only	5	31	0.00 ± 0.00	0.26 ± 0.68	0.03 ± 0.18	0.16 ± 0.73	0.19 ± 0.75
	10	9	0.00 ± 0.00	0.44 ± 1.01	0.00 ± 0.00	0.22 ± 0.44	0.22 ± 0.44
Cyclophosphamide	-	30	1.21 ± 1.37	4.31 ± 5.24	2.79 ± 3.27	9.66 ± 4.07	12.21 ± 6.16
Methotrexate	-	18	1.12 ± 1.17	3.29 ± 3.22	7.59 ± 5.09	1.76 ± 1.60	9.35 ± 5.23

Mean ± S.D were given for all parameters (nuclear aberrations, binucleated cells, and micronucleated erythrocytes), statistically significant differences compared to NaCl controls were only given for cyclophosphamide (*p*<0.001) and methotrexate (*p*<0.001) but not any other treatment regimen (*p*>0.05) as evaluated using one-way ANOVA with Dunnett post-testing.

### Micronuclei test

On day 11, blood from the umbilical artery was collected using a micro-hematocrit capillary (Brand, Germany). Subsequently, blood smears and panoptic May-Grünwald-Giemsa staining were carried out [52]. For each blood smear, 1000 erythrocytes were investigated for the presence of micronuclei, nuclear aberrations, and/or binucleation using an H 500 microscope with 100x oil immersion objective (Helmut Hund, Germany). Erythrocyte staging (Fig 1C) was carried out as previously described [40, 53, 54]. The sum of micronucleated polychromatic and normochromatic definite erythrocytes was obtained and used as an index for genomic toxicity. According to Müller and Streffer [55], a micronucleus is a small nucleus present in addition to the main nucleus. Micronuclei are similar to the morphology of cell nuclei and display a round to oval form with clear borders and their size being up to one third of the diameter of the cell nucleus. Additionally, cells with nuclear aberrations and binucleated cells were counted, as they can be alert parameters for genotoxicity Cells were counted as binucleated if they showed two uniform main nuclei, whereas non-uniform multiple nuclei without a main nucleus were counted as nuclear aberrations, if not fulfilling the criteria for micronuclei [31].

### The antioxidant potential (AOP) in the extra-embryonic compartment

On day 11 and immediately after exposure to control agents or 10 min of plasma treatment (kINPen MED), the inner egg’s membrane was removed and blood was collected into heparinized capillaries (Radiometer Medical, Denmark). Alternatively, collection took place 1 h after plasma treatment to account for a possible delay in oxidative processes. Day 11 was chosen because it is technically challenging to draw blood from the CAM capillaries at earlier time points whereas from day 12 on the embroy’s nervous system develops, making this approach an *in vivo* animal model. The early sampling time point (immediately after treatment and due to experimental handling about 10 min after exposure) was chosen because reactive species quickly react with antioxidants, making a reduced AOP in blood plasma likely. The second blood sampling time point (60 min after exposure) was chosen to investigate late alteration or even recovery of the blood’s AOP after plasma treatment. Drawing blood from the same egg multiple times is not possibility as the veins are very fragile [51]. As positive control, UV-A radiation was generated using a Spectroline EA-160/FE at 50 Hz and 0.17 A (Spectronics Corporation, USA) resulting in a final power of 6 W longwave UV-A at 365 nm (distance: 2 cm). As negative control 0.9% NaCl was applied. Capillaries were gently mixed, centrifuged at 1000 x g for 10 minutes, and the blood plasma was stored at -70°C until analysis. The water-soluble antioxidant potential was determined by measuring the blood plasma’s capability to oxidize exogenously added ascorbic acid using a PHOTOCHEM device (Analytik Jena, Germany) as previously described [56]. The eggs used for this study were not the eggs that were used to assess genotoxicity but were bred independently for this assay.

### Statistics

To statistically compare the results of each, the nuclear aberrations, the number of binucleated cells, the number of micronucleated erythrocytes, and the AOP-values, one-way analysis of variances was used with Dunnett post-testing correcting for multiple comparison with *, **, and *** indicating *p*-values of <0.05, <0.01, and <0.001, respectively. Analysis was carried out using prism 6.07 (Graphpad software, USA).

## Results

### Irritation of the CAM after exposure to different test agents

**Fig 2:**
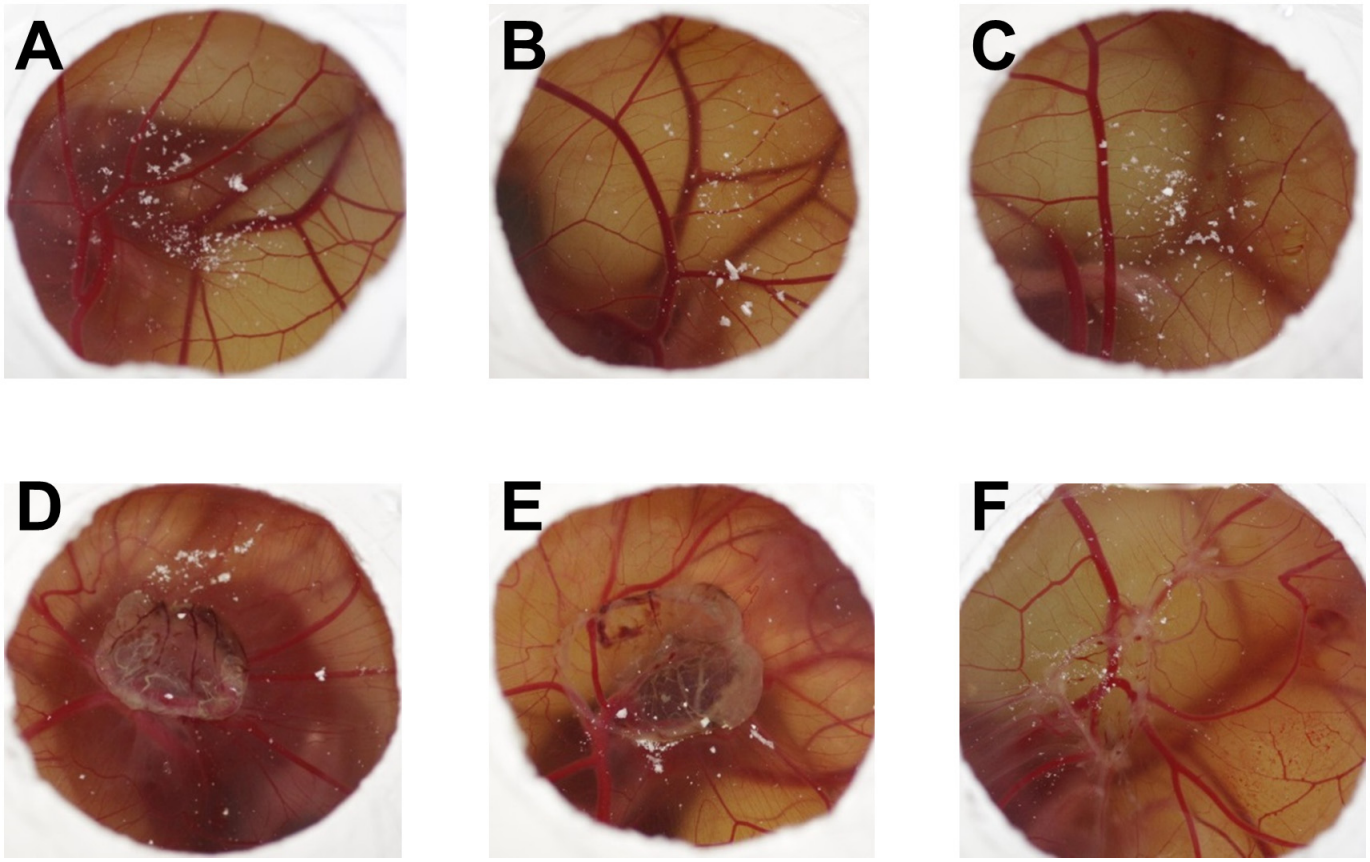
CAM irritation of different test agents. Given are representative images of the CAM three days after exposure to 0.9% NaCl solution (**A**), cyclophosphamide (**B**), methotrexate (**C**), argon gas treatment for 10 min (**D**), kINPen MED plasma treatment for 10 min (**E**), kINPen 09 plasma treatment for 2.5 min (**F**). D-F show hemorrhages, lysis/discoloration, coagulation (thrombus-intravascular and extravascular), and/or increased opacity.

The irritation of the CAM gives a qualitative measure of disturbed tissue owing to exposure to the test agent in question (Fig 2). Three days after exposure, both the negative (0.9% NaCl solution) and the positive (cyclophosphamide and methotrexate) controls did not result in any irritation of the CAM (Fig 2A–2C). In contrast, the physical pressure of the argon gas flow alone caused disturbed tissue pattern on the CAM (Fig 2D). Plasma treatment with both the kINPen MED and kINPen 09 resulted in similar structures (Fig 2E and 2F).

### Evaluation of the HET-MN test model

The HET-MN is a well-established system to test the genotoxicity [40]. The mutagenic agents cyclophosphamide and methotrexate have often been used as positive controls. The application of these substances yielded a mutagenic rate of micronucleated erythrocytes of 12.21 ‰ ± 6.16 ‰ and 9.35 ‰ ± 5.23 ‰, respectively (Table 1). Although thresholds and standard deviations are lab-specific, these results are about similar to what others have found [51, 57]. Nuclear aberrations and the number of binucleated cells in positive controls were significantly increased (*p*<0.001 for cyclophosphamide; *p*<0.001 for methotrexate) compared to both NaCl-treated controls and plasma-treated samples. Test validity was given as well for 0.9% NaCl solution (0.17 ‰ ± 0.38 ‰). The number of micronuclei in NaCl-treated samples (0.30 ‰ ± 0.70 ‰) was very low and comparable with that of double-distilled water in previous reports (1.00 ‰ ± 0.90 ‰) [57].

### Evaluation of the mutagenicity of cold plasma generated by the kINPen

Reactive species are known to be mutagenic at very high concentrations. The plasma of the tested two types of plasma jets is ignited by using argon as feed gas. Accordingly, the argon gas was tested for its genotoxic effects (Table 1). Both treatment times (5 min and 10 min) did not induce an enhanced micronuclei formation (0.19 ± 0.75 ‰ and 0.22 ± 0.44 ‰, respectively). No nuclear aberrations were found in erythrocytes, and the frequency of binucleated cells (0.26 ± 0.68 ‰ and 0.44 ± 1.01 ‰, respectively) was significantly (*p*<0.001) different from positive controls. No exposure to the plasma of the kINPen 09 exceeded the micronuclei formation compared to the negative controls. Nuclear aberrations were only found for 2.5 min of treatment (0.11 ‰ ± 0.31 ‰) but were about 10 fold lower compared to the positive controls. The number of binucleated cells was similar to numbers determined in the cells of the hen’s egg receiving argon gas controls, indicating a negligible influence of the plasma itself. This was also the case for the nuclear aberrations found in erythrocytes following treatment with the kINPen MED. Similar to the kINPen 09, nuclear aberrations were not present, except in the 4 min group (0.07 ‰ ± 0.26 ‰) which was not differing significantly to the NaCl group (*p* = 0.16). Importantly, the number of cells with micronuclei was not significantly different from negative controls for any plasma treatment time (3–10 min). Within these samples, the highest micronuclei frequency was found to be 0.39 ± 0.63 ‰ for 3 min of plasma treatment. To reduce dehydration, NaCl solution (0.9%) was used as a negative control and for moistening purposes before or after the plasma treatment. While in the former regimen, evaporation of the water of the NaCl solution took place, positively regulating the exogenous osmotic pressure, the latter regimen was used to control for this effect. However, similar negative results between both regimes suggest such processes to be of minor importance. This was also true for CAM irritation (data not shown). Comparing the micronuclei frequencies of any plasma treatment mode to the NaCl negative control, no statistically significant difference could be found (*p* = 0.99). In order to eliminate possible negative effects of the kINPen plasma on erythrocytes maturation by potentially affecting the total number of micronuclei being formed, the ratio of polychromatic and normochromic erythrocytes was determined [31]. For the plasma treatment group (10 min), the ratio was calculated to be 0.79 which does not substantially differ from the accompanying negative control (0.74).

### Chicken embryo viability

**Fig 3:**
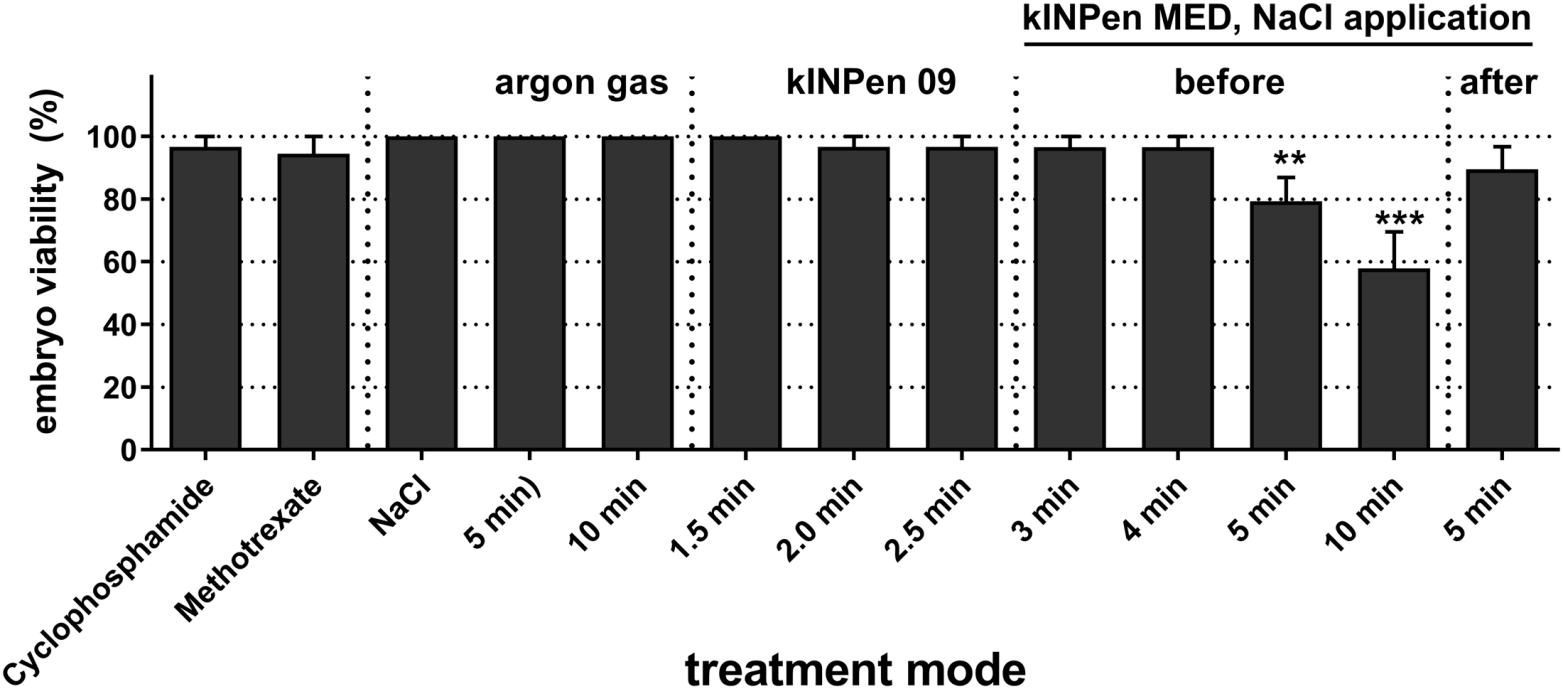
Embryo viability following exposure to test agents. Egg membranes were treated with different test agents. After three days, the embryo viability was determined for each group. Shown are mean values +S.E. of 11 independent experiments. Statistical analysis was performed using one-way ANOVA with Dunnett post-testing.

The chicken embryo viability was determined three days after application of the test agents (Fig 3). Cyclophosphamide and methotrexate showed a very modest toxicity (3–6%) whereas NaCl and argon gas treatment did not cause any chicken embryo mortality. Exposure to the plasma of kINPen 09 demonstrated a similar lethality compared to that of the positive controls. This was also true for HET-MN treatment with the kINPen MED for short exposure times (3 min and 4 min). However, longer exposure for up to 10 minutes caused acute toxicity with mortality rates of 10–42%. There was no strong difference between application of the NaCl solution before or after the plasma treatment.

### Antioxidant potential

**Fig 4:**
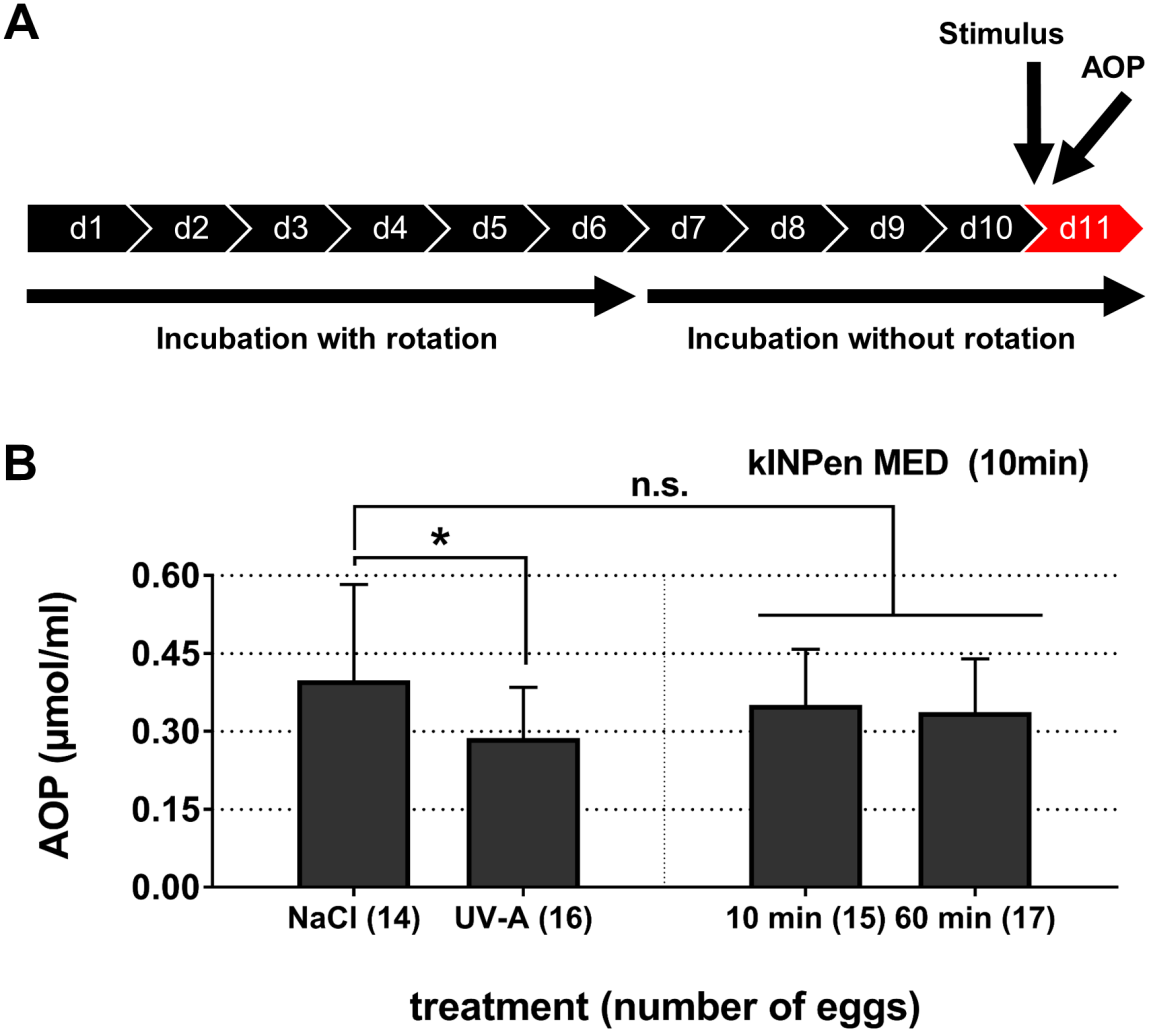
The antioxidant potential in blood plasma. Chicken blood plasma was collected after cold plasma treatment, and the antioxidant potential (AOP) was measured by means of assessing the total ascorbic acid equivalents that have not been oxidized by the sample. (**A**) A experimental scheme is shown for investigating the blood plasma of chicken embryos that were treated with the kINPen MED. (**B**) AOP of blood plasma collected after treatment with 0.9% NaCl solution, UV-A, or kINPen plasma (10 min). UV-A treatment differed significantly (*p*<0.05) from NaCl controls whereas AOP in blood plasma 10 min (*p* = 0.62) or 60 min (*p* = 0.40) after exposure to the cold physical plasma of the kINPen MED did not (mean +S.D.).

As a next step, we sought to determine whether the acute cytotoxic effect of the longer exposure times with the kINPen MED was due to excessive oxidation of the hen’s egg blood components via the plasma-derived reactive components. If such an oxidation effect would have been active systemically, we hypothesized that this should be reflected in the capacity of blood plasma to protect ascorbic acid from experimental oxidation (antioxidant potential, AOP). Irradiation of the inner shell membrane with UV-A significantly (*p*<0.05) decreased the AOP of blood plasma (Fig 4). By contrast, the AOP of plasma-treated embryos differed non-significantly immediately (*p* = 0.62) or 1 h (*p* = 0.40) after exposure when compared to NaCl-treated controls.

## Discussion

For the testing of cytotoxic and/or mutagenic agents, the HET-MN model takes an intermediate position between cell culture experiments and assessing effects in fully developed mammalian organisms, such as rodents. Unlike cell cultures, chicken embryos metabolically (phase I and phase II reactions) convert exogenously added substances [48], making this model more suitable to mimic systemic cytotoxic and/or genotoxic effects in complex organisms. At day eight of incubation, mainly definitive erythrocytes are present, which are formed by embryonic hematopoietic stem cells in the yolk sac [54, 58]. In the latter, biotransformation and erythropoiesis are parallel processes [48]. Up to day 13, the limited functionality of the embryonic spleen disallows a rapid phagocytosis of damaged erythrocytes or micronucleated erythrocytes [59] which makes this model suitable for genotoxic studies [60]. Further, a long exposure time of test agents is recommended to increase the sensitivity of the assay [41]. Thus, application at day eight and incubation to day 11 (72 h) was favored in the present study because it yields higher micronuclei rates, lower embryo toxicity, and higher xenobiotic metabolism [47, 48].

In contrast to red blood cells of mammalian organisms, avian erythrocytes are nucleated [61]. Accordingly, the HET-MN detects structural chromosome fragmentation and numerical aberrations. [57]. Furthermore, it should be noted that DNA repair mechanisms in avian erythrocytes are not as effective as in human cells [62, 63]. However, this can be neglected when interpreting the present results, because no genotoxic effects were evoked by cold plasma. Despite this finding, plasmas treatment adversely affected chicken embryo viability. This clinical impression was reflected by an increasing irritation of the CAM (coagulation, thrombus, and hemorrhage) and an enlarged irritation area as well as an increasing loss of viability. A slight but non-significant decrease of the AOP in the plasma group 10 min and 60 min after exposure underlined this view. In the embryo, lipid oxidation has been detected 9 h following lead injection [64] but this seems to be a rather secondary reaction whereas the plasma-derived ROS are short-lived and their effect on AOP should be apparent swiftly. Both the decrease in AOP and viability suggested that cold plasma components (atomic size: argon 0.21 nm; oxygen 0.12 nm; bond length of O, OH, ^1^O_2_, and NO ~0.1 nm; H_2_O_2_ ~0.34 nm; O_3_ ~0.13 nm) [65] diffused to the embryo through the inner membrane (pore size: 25 nm) [66].

The plasma’s main components, reactive oxygen and nitrogen species, can be detoxified and metabolically controlled by living cells and organisms via glutathione, for example [67]. Experimental supplementation of e.g. catalase protects cells *in vitro* from excessive oxidation induced by plasma [68, 69]. Also, the chicken embryo possesses strong safeguards against oxidative stress, such as ascorbic acid, α-tocopherol, carotenoid, reduced glutathione, catalase, and superoxide dismutase [70–73]. Yet, reactive species are short-lived and antioxidants need to be present locally in order to counteract the plasma-derived ROS. Moreover, the elevated mortality of chicken embryos following 10 min of kINPen MED plasma treatment suggested a limited control of toxic ROS if their concentration had exceeded a certain local threshold to act systemically, although this was not significantly reflected by AOP measurements at the early (10 min) or late (60 min) sampling time points. Hence, this read-out system may have limitations and future studies should collect the remaining liquid on the egg’s membrane or other egg material after plasma treatment to find markers indicative for ROS-stress. Yet, and assuming a treatment area of 2 cm^2^, it should be stressed that the long exposure times used in this study exceeded the therapeutically effective duration for the kINPen 09 (5 s per cm^2^) [49] 15 fold and for the kINPen MED (30–60 s per cm^2^) [46] 5–10 fold. As these treatment regimens did not cause significant genotoxicity they suggest the kINPen plasma not to be a mutagenic hazard within such treatment times.

However, possible limitations of our model need to be stated. Due to their high reactivity and short-lived nature, introduction of ROS/RNS into biological systems does not fit classical parameters such as bioavailability. Moreover, detecting ROS/RNS-mediated oxidation in 3D tissues is highly challenging. This also complicates the AOP-measurements in whole blood as these do not reflect tissue oxidation. Nonetheless, our provided data may add relevance as a more complex test system was used compared to other studies like Comet-assay or *γ*-H2AY assay in cell culture models. As future option, the investigation of red blood cells should be carried out sooner (24 h instead of 72 h) after plasma treatment to identify possible repair mechanism in the HET-MN [41].

## Conclusion

Regardless of the plasma device used (kINPen 09 and kINPen MED) or the treatment time applied (1.5-10 min), no genotoxic effects of the kINPen plasma were found using the HET-MN model. Also, the global antioxidant defense was not significantly challenged following exposure to the plasma. The data provided with the current study seem to confirm the lack of a genotoxic potential, fostering its presumably safe applications in veterinary and human medicine.

## Supporting Information

S1 File Minimal Data Set. (XLSX)
